# Off-label Use of Direct Oral Anticoagulants in Patients Receiving Surgical Mechanical and Bioprosthetic Heart Valves

**DOI:** 10.1001/jamanetworkopen.2021.1259

**Published:** 2021-03-08

**Authors:** Ankur Kalra, Sajjad Raza, Baqir Hasan Jafry, Harley E. King, Joseph A. Lahorra, Lars G. Svensson, Samir R. Kapadia

**Affiliations:** 1 Department of Cardiovascular Medicine, Heart, Vascular, and Thoracic Institute, Cleveland Clinic, Cleveland, Ohio; 2 Heart, Vascular, and Thoracic Department, Cleveland Clinic Akron General, Akron, Ohio; 3 Cleveland Scientific Consulting, Cleveland, Ohio; 4 Aga Khan University, Karachi, Pakistan; 5 Department of Thoracic and Cardiovascular Surgery, Cleveland Clinic, Cleveland, Ohio

## Abstract

This cohort study assesses direct oral anticoagulant use in patients with surgical prosthetic heart valves in the United States and evaluates differences in preoperative and postoperative profiles in patients discharged while receiving direct oral anticoagulant vs warfarin.

## Introduction

In patients with mechanical heart valves, use of direct oral anticoagulants (DOACs) is currently contraindicated, and their use in patients with bioprosthetic heart valves is off-label.^1,2^ We sought to determine the current state of use of DOACs in patients with surgical prosthetic heart valves in the US and evaluate differences in preoperative and postoperative profiles among patients discharged while receiving DOACs vs warfarin.

## Methods

This retrospective cohort study was conducted using data extracted from the Society of Thoracic Surgeons Adult Cardiac Surgery Database risk calculator, version 2.81.^3^ Patients who underwent surgical aortic valve replacement or mitral valve replacement with either mechanical heart valves or bioprosthetic heart valves between July 2014 and June 2017 were included. Data were analyzed from May 1 to September 30, 2020. Patients who were not alive at the time of discharge were excluded. Descriptive analyses were performed to summarize variables. The Cleveland Clinic institutional review board determined this study to be exempt from review owing to use of deidentified data, with institutional-determined waiver of informal consent (oral or written). This study followed the Strengthening the Reporting of Observational Studies in Epidemiology (STROBE) reporting guideline for cohort studies. Statistical analysis was performed using Python, version 3.6.7 (Python Software Foundation) and Microsoft Excel (Microsoft Corp). Statistical significance was set at 2-tailed *P* < .05.

## Results

The study population comprised 177 915 patients; 62% were male and 38% were female. The mean (SD) age of the study population was 62.2 (10.8) years. The use of DOACs was observed among 78.6% (858 0f 1092) hospitals and 59.6% (1627 of 2731) physicians captured in the STS database. In patients undergoing aortic valve replacement with mechanical heart valves (n = 18 142), the overall use of DOACs at discharge over the study period was 1.1% (193 of 18 142; 129 patients received factor Xa inhibitors, and 69 patients received thrombin inhibitors): 1.25% in 2014, 0.99% in 2015, 1.09% in 2016, and 1.17% in 2017 for aortic valve replacement (*P* = .84 for trend) (Figure). In patients undergoing mitral valve replacement with mechanical heart valves (n = 13 942), the overall use of DOACs at discharge over the study period was 1.04% (139 of 13 942; 94 patients received factor Xa inhibitors, and 46 patients received thrombin inhibitors): 1.25% in 2014, 0.91% in 2015, 1.16% in 2016, and 0.93% in 2017 (*P* = .45 for trend). In patients undergoing aortic valve replacement with bioprosthetic heart valves (n = 116 203), the overall use of DOACs over the study period was 4.66% (5625 of 116 203; 4622 patients received factor Xa inhibitors, and 680 patients received thrombin inhibitors), and the use increased over the study period: 3.30% in 2014, 3.80% in 2015, 5.14% in 2016, and 6.64% in 2017 (*P* = .02 for trend). In patients undergoing mitral valve replacement (n = 39 243) with bioprosthetic heart valves, the overall use of DOACs over the study period was 5.89% (2180 of 39 243; 1906 patients received factor Xa inhibitors, and 289 patients received thrombin inhibitors), and the use increased over the study period: 3.94% in 2014, 4.97% in 2015, 5.66% in 2016, and 7.72% in 2017 for mitral valve replacement (*P* = .03 for trend).

**Figure.**
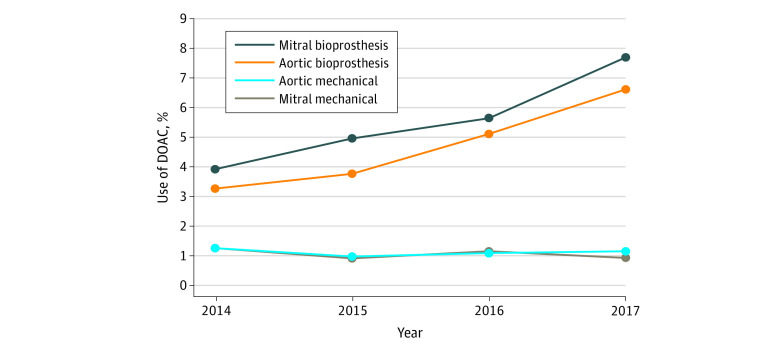
National Trend in Use of Direct Oral Anticoagulants (DOACs) Among Patients With Prosthetic Heart Valves There were 2365 (1.33%) patients who received both aortic and mitral mechanical valves, and 7025 (3.94%) patients who received both aortic and mitral bioprosthetic valves.

In patients receiving aortic valve replacement or mitral valve replacement with mechanical heart valves, 88 patients were discharged receiving exclusively DOAC (no warfarin), and 26 474 receiving exclusively warfarin (no DOAC). Of these, patients discharged receiving DOAC were older (mean [SD] age, 60.8 [12.5] years) compared with those discharged receiving warfarin (mean [SD] age, 53 [11.8] years) (*P* < .001), and there was a greater prevalence of preoperative hypertension (83.0% [73 of 88] vs 68.0% [17 974 of 26 398]; *P* = .003), dyslipidemia (69.3% [61 of 88] vs 57.0% [14 926 of 26 351]; *P* = .02), peripheral arterial disease (18.2% [16 of 88] vs 6.6% [1747 of 26 331]; *P* < .001), heparin-induced thrombocytopenia antibody (14.0% [2 of 14] vs 1.9% [86 of 4490]; *P* < .001), and lower mean (SD) international normalized ratio (1.07 [0.15] vs 1.13 [0.4]; *P* < .001) (Table). Preoperative use of factor Xa inhibitors was significantly higher for patients discharged receiving DOAC than for those discharged receiving warfarin (3.8% [3 of 80] vs 0.3% [73 of 24 721]; *P* < .001). Postoperative (before discharge) events were also higher in patients discharged receiving DOACs than for those discharged receiving warfarin, such as atrial fibrillation or flutter (43.0% [38 of 88] vs 22.0% [5834 of 26 474]; *P* < .001), reoperation for bleeding (9.1% [8 of 88] vs 3.0% [783 of 26 474]; *P* = .001), venous thromboembolism (3.4% [3 of 88] vs 0.5% [138 of 26 474]; *P* = .001), pulmonary thromboembolism (1.1% [1 of 88] vs 0.04% [11 of 26 474]; *P* < .001), and deep vein thrombosis (3.4% [3 of 88] vs 0.4% [112 of 26 474]; *P* < .001).

**Table.** Differences in Preoperative and Postoperative Profiles Among Patients With Prosthetic Heart Valves Discharged Receiving DOACs vs Warfarin

Characteristic	Mechanical					Bioprosthetic				
	DOAC (n = 88)^a^		Warfarin (n = 26 474)		*P* value	DOAC (n = 6740)^b^		Warfarin (n = 48 107)		*P* value
	Total No.^a^	No. (%)	Total No.^c^	No. (%)		Total No.^c^	No. (%)	Total No.^c^	No. (%)	
**Demographic**										
Age, mean (SD) y	88	60.8 (12.5)	26 474	53 (11.8)	<.001	6740	66.3 (7.8)	48 107	65.0 (9.0)	<.001
Female	88	40 (45)	26 474	11 634 (44)	.62	6740	2384 (35)	48 107	19 115 (40)	<.001
Male	88	48 (55)	26 474	14 827 (56)	.65	6740	4353 (65)	48 107	28 980 (60)	<.001
Weight, mean (SD), kg	88	88.2 (24.3)	26 474	88.8 (23.8)	.82	6740	99.6 (22.5)	48 107	88.4 (21.6)	<.001
Height, mean (SD), cm	88	172 (11)	26 474	171 (11.3)	.45	6740	172 (10.6)	48 107	171 (10.8)	<.001
**Preoperative**										
Diabetes	88	26 (29.5)	26 412	6369 (24)	.20	6728	2405 (35.7)	48 025	15 992 (33)	<.001
Hypertension	88	73 (83)	26 398	17 974 (68)	.003	6731	5798 (86.1)	48 035	38 728 (81)	<.001
Dyslipidemia	88	61 (69.3)	26 351	14 926 (57.0)	.02	6717	5135 (76.4)	47 953	34 805 (73)	<.001
Dialysis	88	3 (3.4)	26 418	1210 (4.6)	.46	6726	132 (2.0)	48 039	1555 (3.2)	<.001
Arrhythmia	88	41 (47)	26 368	8023 (30)	<.001	6725	3683 (54.8)	47 947	20 109 (42.0)	<.001
Arrhythmia (atrial fibrillation)	40	36 (90)	7921	6686 (84)	.32	3658	3298 (90)	19 959	17 678 (89)	<.001
Cerebrovascular disease	88	18 (20.5)	26 251	4011 (15.3)	.15	6712	1492 (22.2)	47 829	9422 (19.7)	<.001
Peripheral arterial disease	88	16 (18.2)	26 331	1747 (6.6)	<.001	6714	770 (11.5)	47 906	4941 (10.3)	<.001
Endocarditis	88	6 (6.8)	26 436	3585 (13.6)	.06	6734	511 (7.6)	48 055	4539 (9.4)	<.001
Prior myocardial infarction	85	15 (17.6)	26 301	3294 (12.5)	.13	6701	1286 (19.2)	47 823	8770 (18.3)	<.001
Thoracic aortic disease	88	3 (3.4)	26 270	1791 (6.8)	.17	6687	414 (6.2)	47 862	2645 (5.5)	<.001
Liver disease	88	6 (6.8)	26 234	1318 (5.0)	.35	6674	334 (5.0)	47 766	2312 (4.8)	<.001
Hematocrit, mean (SD)	88	37.6 (6.5)	26 474	38.8 (5.9)	.08	6740	39.2 (5.7)	48 107	38.7 (5.8)	<.001
Platelets, mean (SD)	88	224 598 (75 325)	26 474	225 494 (76 173)	.91	6740	211 596 (70 625)	48 107	214 992 (72 966)	<.001
International normalized ratio, mean (SD)	88	1.07 (0.15)	26 474	1.13 (0.4)	<.001	6740	1.11 (0.21)	48 107	1.15 (0.37)	<.001
Heparin-induce thrombocytopenia antibody	14	2 (14.0)	4490	86 (1.9)	<.001	1022	34 (3.3)	8324	192 (2.3)	<.001
**Preoperative medication**										
Aspirin	87	40 (46)	26 308	11 323 (43)	.49	6691	3556 (53.1)	47 834	24 679 (52)	<.001
Warfarin	81	0 (0)	24 782	369 (1.5)	.22	6323	25 (0.4)	44 934	743 (1.7)	<.001
Adenosine diphosphate inhibitors (within 5 d)	88	2 (2.3)	26 375	386 (1.5)	.41	6707	150 (2.2)	47 955	1018 (2.1)	<.001
Glycoprotein IIb/IIIa inhibitor	88	0 (0)	26 389	44 (0.2)	.51	6711	21 (0.3)	47 992	94 (0.2)	<.001
Factor Xa inhibitors	80	3 (3.8)	24 721	73 (0.3)	<.001	6300	165 (2.6)	44 853	220 (0.5)	<.001
Antiplatelets (within 5 d)	81	2 (2.5)	24 794	446 (1.8)	.50	6323	91 (1.4)	44 974	975 (2.2)	<.001
Thrombolytics	88	0 (0)	26 379	37 (0.14)	.52	6714	10 (0.15)	47 993	42 (0.1)	<.001
Thrombin inhibitors	81	0 (0)	24 784	43 (0.17)	.52	6322	33 (0.5)	44 969	100 (0.2)	<.001
**Postoperative event**										
Kidney failure	88	3 (3.4)	26 474	648 (2.4)	.43	6740	150 (2.2)	48 107	1368 (2.8)	<.001
Atrial fibrillation or flutter	88	38 (43.0)	26 474	5834 (22.0)	<.001	6740	3213 (47.7)	48 107	19 239 (40.0)	<.001
Anticoagulant events	88	2 (2.3)	26 474	275 (1.0)	.21	6740	120 (1.8)	48 107	824 (1.7)	<.001
Reoperation for bleeding	88	8 (9.1)	26 474	783 (3.0)	<.001	6740	176 (2.6)	48 107	1647 (3.4)	<.001
Stroke	88	3 (3.4)	26 474	330 (1.2)	.06	6740	135 (2.0)	48 107	819 (1.7)	<.001
Tamponade	88	0 (0)	26 474	59 (0.2)	.49	6740	8 (0.11)	48 107	60 (0.1)	<.001
Transient ischemic attack	88	1 (1.1)	26 474	58 (0.2)	.06	6740	25 (0.37)	48 107	159 (0.3)	<.001
Venous thromboembolism	88	3 (3.4)	26 474	138 (0.5)	.001	6740	168 (2.5)	48 107	870 (1.8)	<.001
Reoperation for valve dysfunction	88	0 (0)	26 474	48 (0.2)	.50	6740	5 (0.07)	48 107	69 (0.1)	<.001
New dialysis requirement	88	2 (2.3)	26 474	403 (1.5)	.43	6740	89 (1.3)	48 107	888 (1.8)	<.001
Pulmonary thromboembolism	88	1 (1.1)	26 474	11 (0.04)	<.001	6740	25 (0.37)	48 107	124 (0.3)	<.001
Deep vein thrombosis	88	3 (3.4)	26 474	112 (0.4)	<.001	6740	143 (2.1)	48 107	717 (1.5)	<.001

Abbreviation: DOAC, direct oral anticoagulants.

^a^ A total of 67 patients received factor Xa inhibitor and 22 received thrombin-inhibitor.

^b^ A total of 5993 patients received factor Xa inhibitor and 769 received thrombin-inhibitor.

^c^ Number of patients with data available.

In patients receiving aortic valve replacement or mitral valve replacement with bioprosthetic heart valves, 6740 patients were discharged receiving exclusively DOAC (no warfarin), and 48 107 receiving exclusively warfarin (no DOAC). There was a greater prevalence of preoperative arrhythmias (54.8% [3683 of 6725] vs 42.0% [20 109 of 47 947]; *P* < .001), and a lesser prevalence of dialysis (2.0% [132 of 6726] vs 3.2% [1555 of 48 039]; *P* < .001) and heparin-induced thrombocytopenia antibody (3.3% [34 of 1022] vs 2.3% [192 of 8324]; *P* < .001) in patients discharged receiving DOAC. Preoperative use of factor Xa inhibitors (2.6% [165 of 6300] vs 0.5% [220 of 44 853]; *P* < .001) and thrombin inhibitors (0.5% [33 of 6322] vs 0.2% [100 of 44 969]; *P* < .001) was higher in patients discharged receiving DOAC than for those discharged receiving warfarin. Patients discharged receiving DOAC had lesser postoperative (before discharge) events like kidney failure (2.2% [150 of 6740] vs 2.8% [1368 of 48 107]; *P* < .001) and reoperation for bleeding (2.6% [176 of 6740] vs 3.4% [1647 of 48 107]; *P* < .001), but occurrence of postoperative events like atrial fibrillation or flutter (47.7% [3213 of 6740] vs 40.0% [19 239 of 48 107]; *P* < .001), venous thromboembolism (2.5% [168 of 6740] vs 1.8% [870 of 48 107]; *P* < .001), and DVT was higher in patients discharged receiving DOACs (2.1% [143 of 6740] vs 1.5% [717 of 48 107]; *P* < .001).

## Discussion

The main limitation of this study is the lack of follow-up data to compare outcomes of DOACs vs warfarin in patients with prosthetic valves. Despite this limitation, our study suggests a prevailing off-label use of DOACs in patients with prosthetic heart valves without satisfactory safety data. Until the completion of randomized clinical trials that provide sufficient evidence for DOAC use, physicians may wish to exercise caution with regard to DOAC prescription for patients with prosthetic heart valves.
